# Controlling the lodging risk of rice based on a plant height dynamic model

**DOI:** 10.1186/s40529-022-00356-7

**Published:** 2022-08-26

**Authors:** Dong-Hong Wu, Chung-Tse Chen, Ming-Der Yang, Yi-Chien Wu, Chia-Yu Lin, Ming-Hsin Lai, Chin-Ying Yang

**Affiliations:** 1 Crop Science Division, Taiwan Agricultural Research Institute, Council of Agriculture, Taichung, 413008 Taiwan; 2 Graduate Institute of Biotechnology, National Chung Hsing University, Taichung, 40227 Taiwan; 3 Department of Civil Engineering, and Innovation and Development Center of Sustainable Agriculture, National Chung Hsing University, Taichung, 40227 Taiwan; 4 Pervasive AI Research (PAIR) Labs, Hsinchu, 30010 Taiwan; 5 Taichung District Agricultural Research and Extension Station, Council of Agriculture, Taichung, Taiwan; 6 Miaoli District Agricultural Research and Extension Station, Council of Agriculture, Miaoli, Taiwan; 7 Department of Agronomy, National Chung Hsing University, Taichung, 40227 Taiwan; 8 Smart Sustainable New Agriculture Research Center (SMARTer), National Chung Hsing University, Taichung, 40227 Taiwan

**Keywords:** Rice, Nitrogen fertilizer, Plant height, Lodging, Taste quality

## Abstract

**Background:**

Rice is a key global food crop. Rice lodging causes a reduction in plant height and crop yield, and rice is prone to lodging in the late growth stage because of panicle initiation. We used two water irrigation modes and four fertilizer application intervals to investigate the relationship between lodging and various cultivation conditions over 2 years.

**Results:**

Plant height data were collected and combined with aerial images, revealing that rice lodging was closely related to the nitrogen fertilizer content. The aerial images demonstrated that lodging mainly occurred in the fields treated with a high-nitrogen fertilizer, and analysis of variance revealed that plant height was signifi-cantly affected by nitrogen fertilizer. These results demonstrated that rice plant height in the booting stage was significantly positively correlated with the lodging results (r = 0.67) and nega-tively correlated with yield (r = − 0.46). If the rice plant height in the booting stage exceeded 70.7 cm and nitrogen fertilizer was continuously applied, according to the predicted growing curve of plant height, the plant would be at risk of lodging. Results showed more rainfall accumulated in the later stage of rice growth accompanied by strong instantaneous gusts, the risk of lodging in-creased.

**Conclusion:**

The results provide predictions that can be applied in intelligent production and lodging risk management, and they form the basis of cultivation management and response policies for each growth period.

**Supplementary Information:**

The online version contains supplementary material available at 10.1186/s40529-022-00356-7.

## Background

Rice (*Oryza sativa* L.) is a key staple for more than 3.5 billion people globally. The Food and Agriculture Organization of the United Nations has estimated that the demand for rice will continue to increase until at least 2035. On limited arable land, maintaining high and stable rice yields is an effective way to meet the world’s growing demand for food (Pan et al. 2019). However, with the intensification of climate change, the frequency and intensity of droughts and heavy rains have increased, leading to increased uncertainty in food production and exacerbating food security problems (Ho et al. 2018; Wu et al. 2018). The occurrence of strong winds, typhoons, and heavy rains during extreme weather events increases the frequency of crop lodging. Lodging limits the yield and quality of rice and is a major concern for many farmers (Liu et al. 2018; Zhao et al. 2019). In the natural environment, lodging occurs when the upper part of the plant increases in weight because of rainfall interception during heavy rains or is unable to tolerate strong winds (Shrestha et al. 2020; Zhao et al. 2019). Cereal crop lodging can be divided into two types: root lodging and stem lodging. Root lodging occurs when the anchorage of the roots in the ground fails and the intact stem tilts, and stem lodging is caused by the development of taller stem nodes in plants; the bending pressure of higher internodes is too high, causing the lower culm internodes to bend or break (Shah et al. 2019). Rice lodging destroys the rice canopy, leading to a decline in photosynthesis capacity and dry matter production, ultimately reducing rice yield and quality. In addition, lodging may have other indirect chain effects, such as rice varieties with weak seed dormancy. After the grains on the panicle make contact with the ground, humidity increases and the seeds germinate or plants collapse, causing difficulties for harvest operations, such as increased demand for grain drying, decreased harvesting efficiency, or increased production costs (Islam et al. 2007; Liu et al. 2018).

Studies have revealed that in addition to genetic differences between varieties or weather factors, the occurrence of lodging is closely related to many external factors such as cultivation methods or nitrogen fertilizer application (Shrestha et al. 2020). Nitrogen fertilizer is one of the main factors affecting rice production, and its proper management is essential for improving the yield of grains. An increase in the amount of nitrogen fertilizer applied leads to a corresponding increase in plant height and biomass. The reasonable application of nitrogen fertilizer can increase rice yields, but its excessive application increases the height of the center of gravity of the crops and reduces the distance between basal internodes. The increased diameter of the stem and thickness of the cell wall results in poor lodging resistance (Sun et al. 2018; Zhang et al. 2019). When application rates of nitrogen fertilizer are increased, the lodging index first decreases and then increases, demonstrating that under moderate nitrogen application, the risk of lodging in rice plants is at its smallest (Zhang et al. 2016).

In recent years, high-throughput plant phenotyping technology based on drone images has matured. This technology allows high-throughput plant phenotypes related to plant height to be used as indicators of yield, carbohydrate storage, and lodging sensitivity (Chou et al. 2020; Han et al. 2018a, 2018b; Holman et al. 2016; Yang et al. 2020a, 2017, 2020b). However, the relationship between plant height and lodging risk is not yet fully understood. In addition, rice is prone to collapse and damage during rainy and typhoon seasons at the time of the first crop in Taiwan, resulting in a decline in rice yield and quality. Therefore, this study focused on the correlation between rice plant height and lodging and rice yield and quality. Through fertilizer control and irrigation water management and supplemented by aerial image analysis technology, we were able to obtain early plant height development information. We aimed to quantify farmers’ years of field management experience to provide them with a reference that enables them to respond to changing climatic and environmental conditions.

## Methods

### Plant materials and experimental field management

In this study, we used the Tainong 71 (TNG71) variety of early maturing *Japonica* rice. This variety is characterized by its taro flavor, excellent rice quality, and it has been ranked among the top 10 recommended varieties of rice in Taiwan. The experiment was conducted in a cultivation test field (24.10° N, 120.41° E) at the Agricultural Experiment Institute of Wufeng District, Taichung City, Taiwan. Two water management modes were designed: conventional plant (CP) and alternate wet and dry (AWD). The main area was divided into four plots for the application of four nitrogen fertilizer levels: (80 (N1), 120 (N2), 160 (N3), and 200 (N4) kg/ha), and each plot had an independent water inlet to avoid the mutual interference of the fertilizers. The area of each cultivation plots was 97.60 m^2^ (8.0 m × 12.2 m) and the row spacing for rice cultivation was 30 cm × 21 cm, and water management was implemented from day 20 after the transplantation of seedlings. A water height of 3–5 cm was maintained in the CP mode throughout the growth period, except for during the field drying period of the booting stage. For the AWD mode, water was supplied to a height of 3–5 cm during the cultivation period, and then irrigation was stopped. After the water level dropped to 0 cm, irrigation began again the following day until a water height of 3–5 cm was reached; this cycle was repeated. To maintain the water level at a height of 1–5 cm during the heading stage, the water supply was continued until the plant reached the mature stage and stopped 5 days before harvest. A microclimate weather station (Demeter Technology Inc ESP-4WPB001) was installed in the cultivation field to collect climate data during the experimental period.

### Investigation of characteristics and evaluation of yield

Each plot contained five plants as the survey unit, and plant height was measured once a week on the 11th and 19th day after transplantation over 2 years (2019 and 2020); the plot yield, yield components, and rice quality were analyzed after harvesting. To obtain the plot yield, 100 plants from the plot were mechanically threshed. After winnowing once, the dry grain weight and moisture content were recorded; then, the yield per unit area was estimated based on the moisture content of 13% for grains. Yield components were sampled from four plants per plot to investigate the number of panicles, seed rate, number of grains per panicle, and thousand grain weight. Rice quality was measured in terms of protein, amylose, and water content using the Kett NIRT Grain Tester AN-820. A rice appearance quality tester (TPMZ-A) was used to analyze the intact rice rate and chalk content, and a rice hardness and viscosity meter (SATAKE RHS1A, RHS1B) was used to test the appearance, hardness, viscosity, and balance of rice.

### Unmanned aerial vehicle photography and image analysis

An Unmanned aerial vehicle (UAV) model was a DJI M200 equipped with a multispectral camera with 20 million pixels (Micasense Rededge-M, MicaSense Inc., Seattle, WA, USA) was used to obtain images in the shutter priority mode, and the shooting time and weather data were recorded, so that at the end of the shoot, the image could be manually checked to confirm whether it was blurred, overexposed, or damaged. The flight height was set at 40 m, and the image resolution was approximately 3.0 cm/pixel. Finally, Agisoft Metashape software was used to perform image stitching with an image overlap rate of at least 80%; the stitched output image was used in image analysis to estimate the lodging percentage. The lodging classification model, which incorporated UAV-image-derived digital surface model and texture information, was analyzed as previously described (Yang et al. 2017).

### Statistical analysis

R software v. 3.6.1 (R Core Team 2019) was used for statistical analyses. Analysis of variance (ANOVA) and "corrplot" functions were used on different days for the correlation analysis of plant height, lodging, and yield (Wei et al. 2017). In addition, the ggplot2 suite (Villanueva and Chen 2019) was used to plot the trend curve of plant height development and lodging.

## Results

### Interpretation of lodging areas in rice fields using aerial photography

**Fig. 1:**
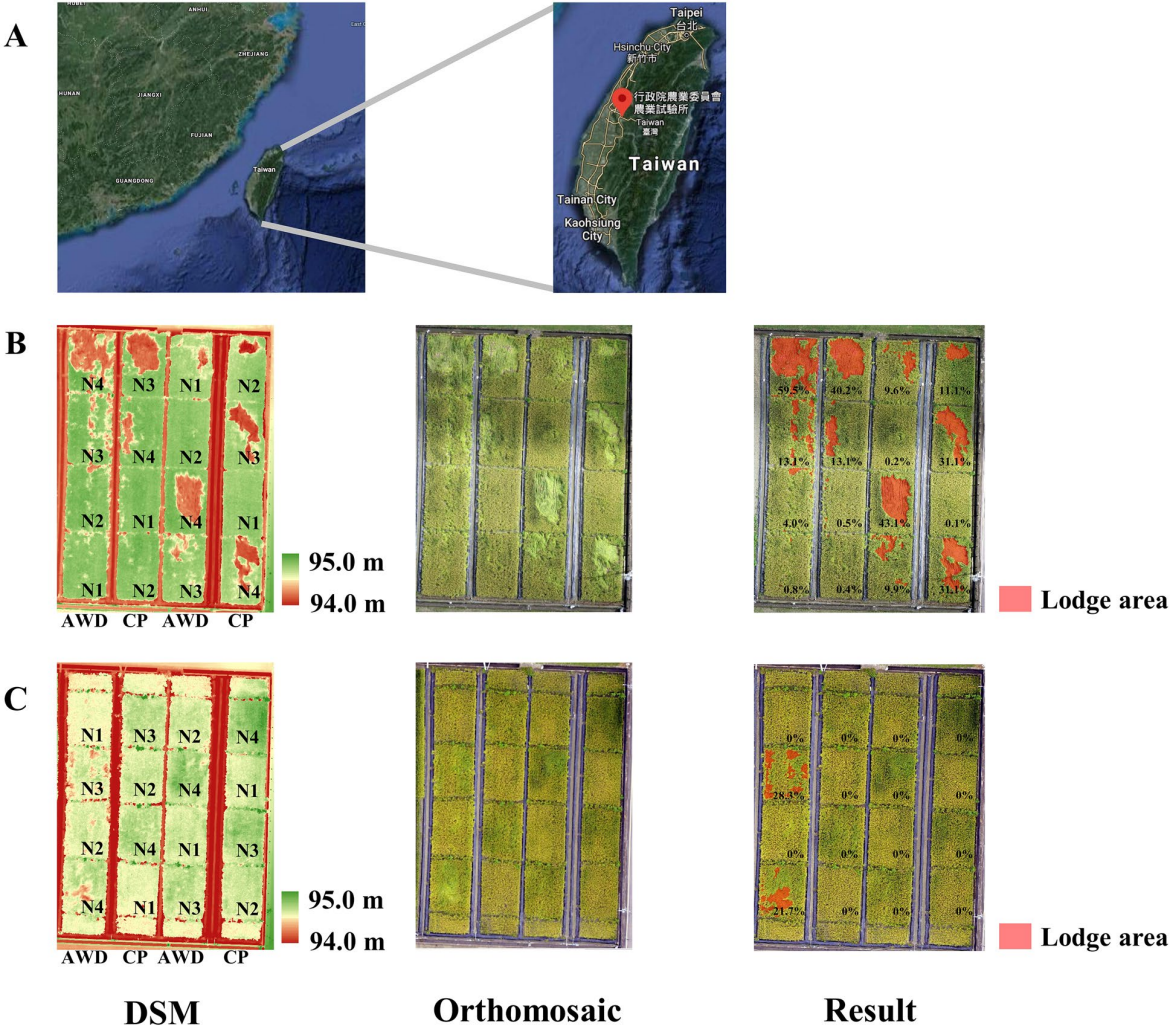
Geographical location of the experiment field and aerial lodging image of TNG 71. **A** Rice field trials were conducted in the central region of Taiwan in 2019 and 2020. **B** Aerial lodging image for 2019. **C** Aerial lodging image for 2020. The digital surface model (DSM) diagram indicates the height difference of the field area; the green block represents the higher area, and the red block represents the lower area. The orthomosaic image is spliced from aerial images. The resulting image is a combination of the DSM image and orthomosaic image; the red block represents the lodging area, and the value represents the lodge area rate. N Fertilizer treatment level is divided into 80 (N1), 120 (N2), 160 (N3), and 200 (N4) kg/ha. *AWD* alternative wet and dry cultivation. *CP* conventional plant cultivation

Rice field trials in the experiment were conducted in central Taiwan for 2 consecutive years (Fig. 1A), and an unmanned aerial vehicle was used to take multispectral images of the rice variety TNG71. First, a digital surface model (DSM) was used to express the spatial distribution of actual topographic features to the height difference of rice plants from the ground to be estimated, and orthomosaic images displayed the distribution of plants on the ground. The two images were then superimposed to evaluate the TNG71 lodging percentage under different types of water management (CP and AWD) and under different fertilizer levels (four nitrogen fertilizer levels). The results revealed that, in 2019, under CP management, both the fertilization rate and lodging area of each plot increased under different fertilization levels, and under AWD management, a lodging percentage of more than 40% occurred at the fertilization rate of 200 kg/ha (N4) (Fig. 1B). In 2020, the N3 and N4 nitrogen level areas exhibited more than 20% lodging percentages under AWD management (Fig. 1C). Our results revealed that the application of excessive nitrogen fertilizer might increase the occurrence of rice lodging.

### Incidence of rice lodging in relation to field moisture, fertilizer management, and meteorological factors

Rice growth is affected by different field management methods and external environmental factors, which can lead to the occurrence of rice lodging. To understand the relationship between rice lodging and different types of water and fertilizer management, the rice lodging percentage, water management, and fertilizer application were analyzed for variance. The results demonstrated that water management and fertilizer application had a significant positive effect on the lodging percentage in 2019, but no significant positive effect was identified in 2020 (Table 1).

**Table 1:** Analysis of variance for lodging in 2019 and 2020

Year	Source of variation	Degree of freedom	Sum of squares	Mean square	*F*	*p-*value
2019	Replucate	1	629.1	629.1		
	Water	1	5.42	5.42	0.063	0.844
	Error1	1	86.63	86.63		
	NF	3	1451.0	483.7	9.823	0.0099^**^
	Water^*^NF	3	1459.9	486.6	988.3	0.0098^**^
	Error2	6	298.4	49.2		
2020	Replucate	1	170.3	170.3		
	Water	1	170.3	170.3	1	0.5
	Error1	1	170.3	170.3		
	NF	3	174.5	58.17	1	0.455
	Water^*^NF	3	174.5	58.17	1	0.455
	Error2	6	349.0	58.17		

^**^values are significant at *p* < 0.01

NF: nitrogen fertilizer

**Fig. 2:**
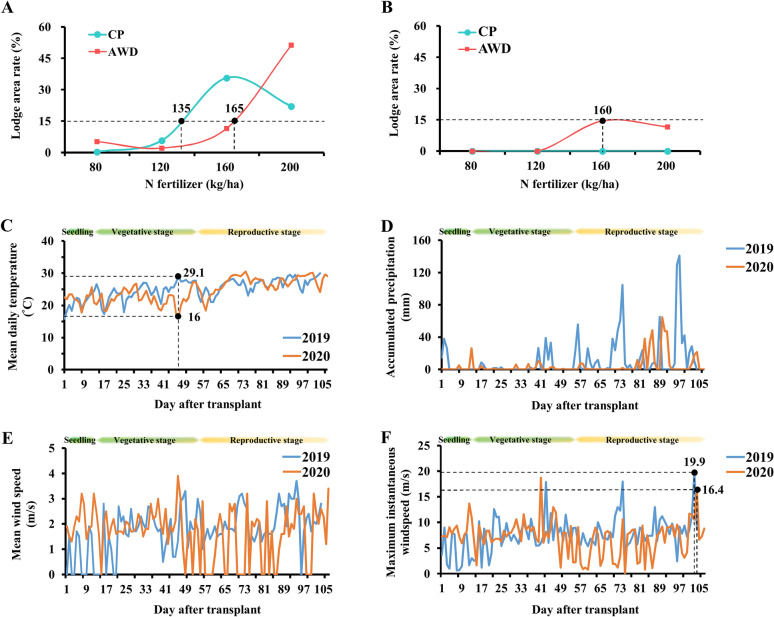
Lodging percentage and meteorological data for TNG71 under different types of water management and levels of fertilizer application. **A** Lodging percentage in 2019. **B** Lodging percentage in 2020. **C** Daily mean temperature in 2019 and 2020. **D** Changes in accumulated precipitation in 2019 and 2020. **E** Mean wind speed in 2019 and 2020. **F** Maximum instantaneous wind speed in 2019 and 2020. The lodging area rate is the lodging percentage in the field. Day after transplantation was calculated from the time the seedlings were transplanted to the field. N Fertilizer treatment level is divided into 80, 120, 160, and 200 kg/ha. *AWD* alternative wet and dry cultivation. *CP* Conventional plant cultivation

In 2019, under CP management, more than 135 kg/ha of fertilizer was applied, whereas under AWD management, more than 165 kg/ha of fertilizer was applied, and a lodging percentage of more than 15% occurred in this area (Fig. 2A). In the first phase of 2020, under AWD management, the lodging percentage exhibited an upward trend when the fertilizer application reached more than 120 kg/ha (Fig. 2B). To explore whether meteorological factors affected the lodging percentage of rice, the data from the weather station were used to analyze the changes in temperature, rainfall, and wind speed over the 2-year study period. The results revealed that the mean daily temperature in the maximum tillering stage of the rice (47 days after transplantation; DAT47) was 29.1 °C in 2019. However, the mean daily temperature in the same growth stage in 2020 was 16 °C, which was 13.1 °C lower than that in 2019 (Fig. 2C). The accumulated precipitation during the rice-growing period in the first phase of 2019 was 1168 mm, which was higher than the accumulated precipitation in the first phase of 2020 (450.5 mm). The accumulated precipitation in 2019 in the 10 days before harvesting was 410 mm, which was 9 times higher than that in 2020. We speculated that this was caused by continuous rainfall during the rice harvest period, and the lodging results were more severe in 2019 than in 2020 (Fig. 2D). According to the meteorological data in 2019, the average wind speed in the 10 days before harvest was 2.01 m/s, and the instantaneous maximum wind speed during the same period was 19.9 m/s. The average wind speed in the 10 days before harvest in 2020 was 1.71 m/s, and the instantaneous maximum wind speed during the same period was 16.4 m/s (Fig. 2E and F). Our results indicated that the higher lodging percentage of rice in 2019 was probably caused by the strong winds and heavy rains during the rice harvest period.

### Correlation between plant height and lodging risk in rice

**Fig. 3:**
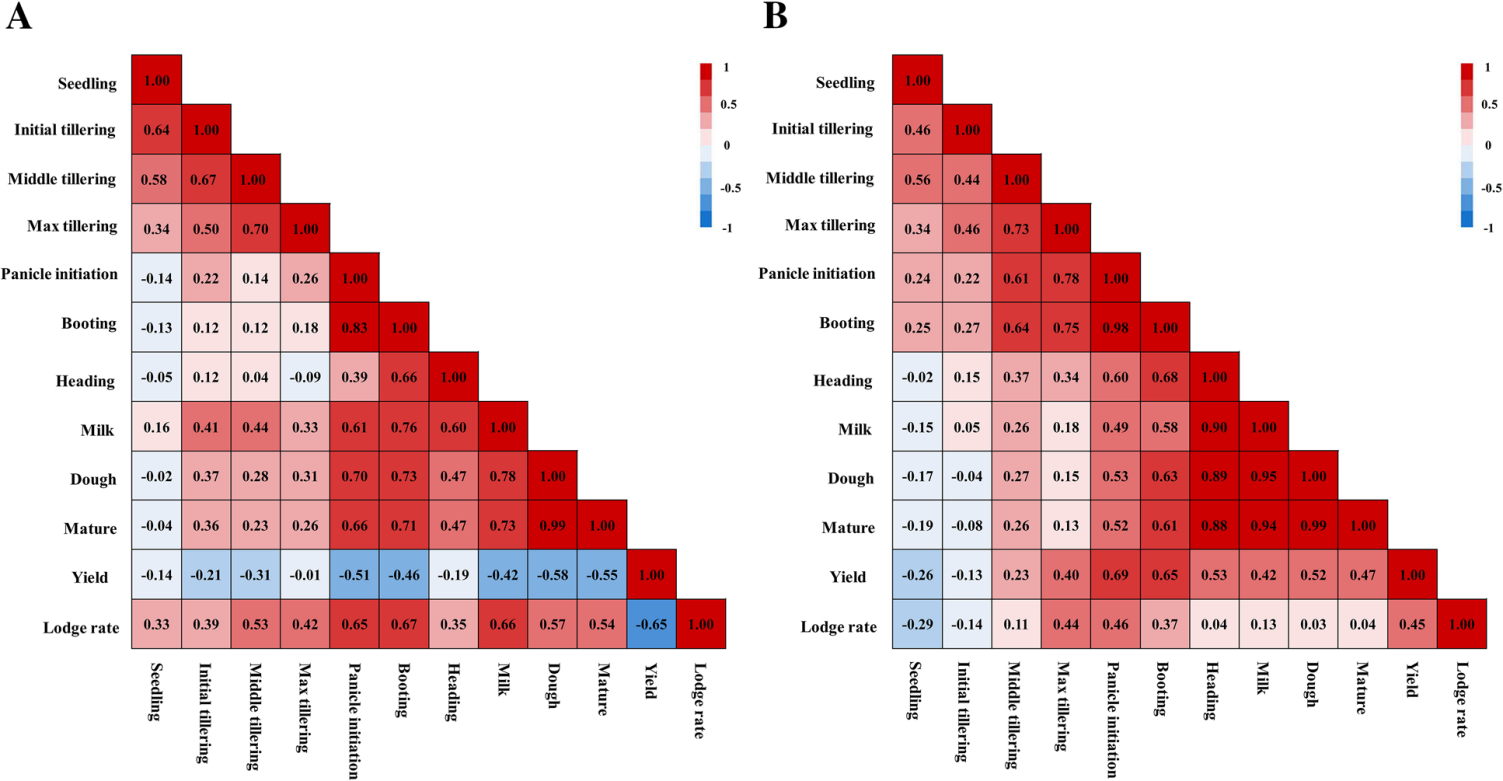
Pearson correlation results for rice plant height, yield, and lodging result in different growth stages. **A** Experiment results in 2019. **B** Experiment results in 2020. The lodging rate is the lodging percentage in the field. The blue blocks represent negative correlations, and the red blocks represent positive correlations

To evaluate the correlation between plant height and lodging risk during rice development, we further observed plant height growth during the whole growth period of TNG71. Our results revealed a significant correlation between rice plant height and nitrogen fertilizer levels in 2019 and 2020, and the interaction effect on plant height was significant for water management and nitrogen fertilizer levels in the middle tillering, maximum tillering, booting, heading, milk, dough, and mature stages of rice in 2019 (Table 2). The relationship between plant height and lodging and yield in different growth stages of rice was further analyzed. Plant height in the booting stage in 2019 was 72.2 ± 2.9 cm (Additional file 1: Table S1), and the Pearson correlation results revealed that plant height was positively correlated (r = 0.67) with lodging and negatively correlated (r =− 0.46) with yield (Fig. 3A). Plant height in the booting stage in 2020 was 62.5 ± 3.6 cm and had no significant positive correlation with lodging (r = 0.37) but was highly positively correlated with yield (r = 0.65) (Fig. 3B). Additional file 1: Table S1 presents the plant heights in different growth stages in 2019 and 2020. Additional file 1: Table S2 These results indicated that the difference in plant height in the booting stage between 2019 and 2020 was a key factor in subsequent rice lodging.

**Table 2:** Analysis of variance for TNG71 plant height in different rice growth stages in 2019 and 2020

Year	Source of variation	Growth stage									
		Seedling	Initial tillering	Middle tillering	Maximum tillering	Panicle initiation	Booting	Heading	Milk	Dough	Mature
2019	Water	3.20	0.003	0.02	0.02	0.12	0.90	3.80	1.21	14.06	21.62
	NF	0.42	0.92	2.63^*^	2.74	19.89^*^	23.50^*^	13.7	9.30	53.00^*^	47.13^*^
	Water^*^NF	0.15	3.25	3.62^*^	0.58	4.92	15.42^*^	15.73	17.60^*^	22.36	23.35
2020	Water	3.63	1.09	5.05	2.65	8.05	0.85	7.80	68.10	92.59	101.61
	NF	0.65	2.35	3.06	7.65	53.03^*^	53.18^*^	26.05	76.47^*^	181.22^*^	202.77^*^
	Water^*^NF	3.29	1.94	1.83	1.40	9.74	7.59	1.61	6.60	28.21	34.86

^*^values are significant at *p* < 0.05

NF: nitrogen fertilizer

**Fig. 4:**
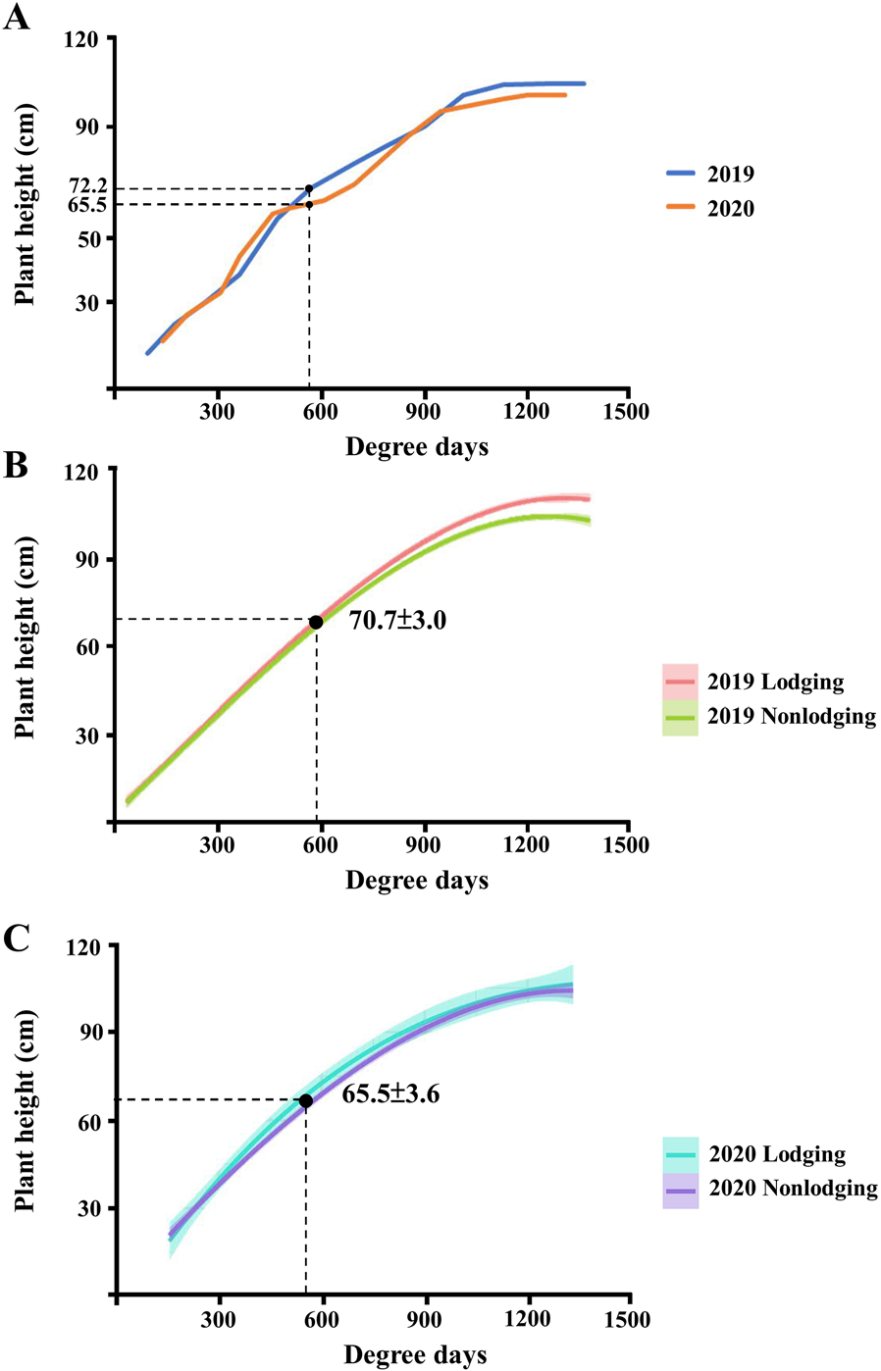
Plant height development of TNG71 in 2019 and 2020. **A** Plant height development of rice in 2019 and 2020. **B** Plant height development of lodging and nonlodging rice in 2019. **C** Plant height development of lodging and nonlodging rice in 2020. The growing degree day is the average of the daily maximum temperature and minimum growth critial temperature (10 °C)

Comparing the continuous changes in the plant height of rice in 2019 and 2020, we noted that the growing degree of rice in the booting stage was 588.9 °C, corresponding to plant heights of 72.2 and 65.5 cm in 2019 and 2020, respectively. The difference in plant height may be related to temperature, rainfall, and other environmental factors. The high-temperature environment and abundant rainfall in 2019 enabled faster plant growth, producing more plant height than that in 2020 in the same growth stage (Fig. 4A) and leading to the occurrence of lodging in the later stage of growth. The difference in plant height between the lodging and nonlodging rice areas in 2019 was analyzed. The results demonstrated that if the rice plant height exceeds 70.7 ± 3.0 cm in the booting stage and sufficient nitrogen fertilizer continues to be applied, lodging risk increases in line with the predicted plant height growing curve. Compared with the plant height in 2019, the plant height in 2020 was only 65.6 cm in the booting stage, which may explain why lodging was less severe in 2020 (Fig. 4B and C).

### Effect of lodging on rice yield and taste quality

The application of nitrogen fertilizer can increase the spikelet and panicle number and enhance rice yield (Kamiji et al. 2011; Xiong et al. 2013). In the present study, ANOVA was performed on our rice yield data, fertilizer content, water use efficiency, and lodging results to evaluate whether lodging affected rice yield. In 2019, rice lodging occurred more severely in the field with high levels of nitrogen, and the nitrogen fertilizer content was significantly correlated with the lodging results and final yield, suggesting that both lodging and yield were affected by the nitrogen fertilizer content. In 2020, lodging occurred less severely than in 2019. Nitrogen fertilizer did not exert a significant positive effect on lodging, but its relationship with panicle number, grain number, fertility, and yield were significant, revealing the positive effects of nitrogen fertilizer on rice yield (Table 3).

**Table 3:** Analysis of variance for TNG71 yield components in 2019 and 2020

Year	Source of variation	Degree of freedom	Water use efficiency	Lodge	Panicle number	Grain number (per Panicle)	Fertility	1000-grain weight	Yield
2019	Replicate	1	0.0005	1.334	28.2	77.9	0.6	1.7	286,503
	Water	1	0.0203	9.95	15.5	116.1	41.6	1.9^**^	175,758
	Error1	1	0.0014	71.32	1.4	4.5	19.4	0.0	15,131
	NF^a^	3	0.0037^*^	1073.5^*^	5.5	37.1	25.2	0.4	1,202,947*
	Water^*^NF^a^	3	0.0034^*^	487.6^*^	37.2	15.7	3.4	0.3	1,469,086**
	Error2	6	0.0008	62.3	9.5*	44.2	8.3	1.1	284,005
2020	Replicate	1	0.0049	170.3	5.4	90.5	30.6	0.2	2943
	Water	1	0.441	170.3	14.5	122.3	69.6	17	456,435***
	Error1	1	0.182	170.3	2.1	44.7	1.9	0.0	4
	NF^a^	3	0.002375^***^	58.17	47.36^***^	86.23^***^	28.01^*^	2.2	780,030***
	Water^*^NF^a^	3	0.0013833^**^	58.17	50.94^***^	178.51^***^	46.15^**^	1.1	404,575**
	Error2	6	0.0002	58.17	4.0	7.8	7.6	0.7	78,514

^*^ *p* < 0.05; ^**^ *p* < 0.01; ^***^ *p* < 0.001

^a^*NF* nitrogen fertilizer

In addition to rice yield, taste quality is a key indicator of rice production. Rice is mainly composed of starch, protein, fat, and minerals. The taste quality of rice is affected by physical and chemical properties, including amylose and protein content, hardness, and cohesion (Aoki et al. 2017; Tran et al. 2005). ANOVA revealed that the application of nitrogen fertilizer had a significant positive effect on the lodging rate, taste, and protein and amylose content in 2019, indicating that lodging caused by a high nitrogen content might change the palatability and protein and amylose content of rice. The 2020 results indicated that water efficiency and nitrogen fertilizer application had a significant positive effect on the appearance, hardness, and taste quality of rice, indicating that these characteristics are also affected by fertilizer and water content (Table 4).

**Table 4:** Analysis of variance for TNG71 rice quality in 2019 and 2020

Year	Source of variation	Degree of freedom	Water use efficiency	Lodge	Chalk	Refined grains	Appearance	Hardness	Cohesion	Balance	Taste value	Palatability	Protein	Moisture	Amylose
2019	Replicate	1	0.0005	1.334	37.7	250.0	0.6	0.0	1.1	0.6	25.0	60.1	2.1	0.10	0.5
	Water	1	0.0203	9.95	60.2	85.7	0.3	0.0	0.7	0.4	13.7	3.7	0.1	0.02	0.2
	Error1	1	0.0014	71.32	20.6	128.2	2.0	0.7	1.0	1.8	73.1	33.1	0.8	0.05	0.0
	NF^a^	3	0.0037*	1073.5*	5.9	122.9	0.9	0.2	1.1	1.0	39.9	57.2****	1.8****	0.01	0.2*
	Water*NF^a^	3	0.0034*	487.6*	69.4	56.6	0.6	0.1	0.6	0.7	26.0	9.6**	0.3**	0.12	0.4**
	Error2	6	0.0008	62.3	29.4	127.1	0.7	0.2	0.6	0.7	28.1	1.6	0.1	0.01	0.1
2020	Replicate	1	0.0049	170.3	0.1	3.2	0.39	0.16	0.0	0.28	8.56	0.4	0.0	0.01	0.0
	Water	1	0.441	170.3	17.3	0.1	0.23	0.1225****	0.2	0.28	11.06	5.4	0.2	0.01	0.0
	Error1	1	0.182	170.3	0.1	0.1	0.02	0.00	0.1	0.02	0.95	4.7	0.1	0.01	0.0
	NF^a^	3	0.002375***	58.17	15.1	11.1	0.7956*	0.21583**	0.6	0.7723*	30.23*	6.2	0.2	0.01	0.0
	Water*NF^a^	3	0.0013833**	58.17	8.2	4.0	0.7223*	0.1425**	0.8	0.734*	29.17*	6.1	0.2	0.01	0.1
	Error2	6	0.0002	58.17	21.4	26.4	0.19	0.03	0.4	0.21	8.82	2.4	0.1	0.01	0.0

^*^ *p* < 0.05; ** *p* < 0.01; *** *p* < 0.001; **** *p* < 0.0001

^a^*NF* nitrogen fertilizer

## Discussion

### Effect of environmental factors on the degree of lodging

Rice stalks support the growth of inflorescence. The insufficient strength of rice stalks can cause plant lodging, which in turn negatively affects grain growth and increases the time required for the harvest (Corbin et al. 2016). During the grain-filling period of barley, accumulated rainfall of up to 300 mm caused plant lodging, resulting in grain damage and reduced yield (Schelling et al. 2003). In this study, we collected weather data from the Central Meteorological Bureau of the Ministry of Communications (Agricultural Experiment Station) for 2019 and 2020 to clarify the relationship between environmental factors and the rice lodging rate. At DAT47, the average daily temperature in 2019 was 13 °C higher than that in 2020, and rainfall in 2019 was also more abundant than that in 2020. The high-temperature and humid environment facilitated faster plant growth, which may explain the obvious difference in plant height in the booting stage between 2019 and 2020. In 2019, heavy rainfall occurred during the10 days before harvesting, and the accumulated rainfall was approximately 10 times higher than that in the same period in 2020. Simultaneously, strong winds with an instantaneous maximum wind speed of 19.9 m/s occurred. Based on this weather data, we speculated that the different percentages of lodging in 2019 and 2020 were caused by the different plant heights resulting from the different amounts of rainfall in the middle growth stage and heavy rainfall in the later growth stage in 2019 (Fig. 2).

### Predicting the final lodging percentage in relation to water management and nitrogen fertilizer application during rice cultivation

Lodging is caused by factors such as the fertilizer concentration and rainfall. An increase in the amount of applied fertilizer can increase rice tillering, plant height, and shoot weight. Under high-nitrogen treatment, the production of sclerenchyma cells and vascular bundle cells in the secondary cell wall of plants is reduced, resulting in fragile stems, which are susceptible to heavy rain, leading to rice lodging (Weng et al. 2017; Zhang et al. 2014). In this study, lodging mainly occurred in the high-nitrogen treatment groups (N3 and N4), and the ANOVA results indicated that the lodging percentage was significantly related to the amount of nitrogen fertilizer applied (Table 1). The application of nitrogen fertilizer directly affects plant height, thereby influencing the risk of lodging. The plant heights in 2019 in groups N1–N4 were 58.7, 61.8, 63.3, and 63.6 cm, respectively, and the corresponding final lodging percentages were 2.75%, 3.93%, 23.58%, and 36.70%, respectively. We speculated that plant height between 61.8 and 63.3 cm in the panicle initiation stage might be key for determining lodging under the external environmental conditions of 2019 (Additional file 1: Table 2). In addition, comparing plant height in the panicle initiation stage in 2019 and 2020, we found that even though plant height in N4 in 2020 reached 67.98 cm, the final lodging percentage was only 5.8%; thus, although plant height reached the lodging threshold, the rainfall before harvesting in 2020 was not as much as that in 2019, indicating that weather conditions are another key factor in rice lodging (Fig. 2D). According to the 2019 results, plant height can be predicted in the early growth stage of rice and can indicate lodging risk. If plant height was 70.7 cm in the booting stage, the application of nitrogen fertilizer should be carefully controlled to prevent the plant from lodging, which is caused by excessive plant height. The accumulated concentration of nitrogen fertilizer should be monitored to help control plant height. If the fertilizer concentration of the field exceeds the threshold amount of fertilizer (78.8 kg) before the booting stage, water management is another effective method to control the plant height of rice. AWD cultivation and reduced nitrogen fertilizer application can maintain the growth curve of rice at a level that avoids lodging. Different cultivation and management methods can be adopted in different stages to reduce the risk of lodging.

Data on fertilizer application rates, plant height, meteorology, and lodging percentage were collected in this study, which can be used by farmers or in future research for evaluating the risk of rice lodging. Farmers can evaluate the plant height of rice in various growth stages to assess the risk of lodging and then reduce or increase the amount of fertilizer used; from the late growth period to the harvest period, farmers should pay attention to meteorological data, particularly to reports of heavy rain or strong winds, and should make the necessary adjustments to avoid the occurrence of lodging.

### Correlation between lodging and rice yield and eating quality

The yield components of rice consist of the number of ears per unit area, number of spikelets per ear, seed setting rate, and average thousand grain weight, and the components of rice quality consist of milling quality, appearance, cooking and eating quality, and rice entrance quality (Ho et al. 2012). Our 2019 results revealed that under CP management, more than 135 kg of nitrogen fertilizer was applied, and under AWD management, more than 165 kg was applied (Fig. 2), leading to a final average plant height of more than 104.5 ± 4.0 cm in the mature stage, which tended to result in lodging. Studies have noted that when rice lodging occurs during the grain-filling period, yield, rice milling quality, milled rice rate, and change in the starch synthesis rate in the grain are reduced, resulting in increased chalkiness (Lang et al. 2012). Although correct nitrogen fertilizer application can improve rice yield, excessive nitrogen fertilizer application increases the risk of lodging, which reduces the final yield. Furthermore, the overapplication of nitrogen fertilizer changes the composition of rice, which may lead to a decrease in rice quality and taste (Gu et al. 2015; Liang et al. 2021). Therefore, the prudent use of nitrogen fertilizer is key for controlling the final rice yield and quality (Tables 3 and 4).

Plant height is a key external trait of rice, which can affect lodging, biomass, and yield. Environmental factors such as temperature, accumulated rainfall, and wind speed during rice growth can affect plant growth. Under continuous rainfall or high wind speeds, the risk of plant lodging is positively correlated with plant height. In this study, the correlation among plant height, lodging, yield, and rice quality was analyzed. Farmers can use the plant height development data and the cultivation management information on fertilizer use and water supply presented in this study to reduce the risk of lodging.

## Supplementary Information

**Additional file1**: **Table S1**. Plant height of TNG71 in different growth stages in 2019 and 2020. **Table S2**. Lodge rate, yield, and plant height of TNG71 under four nitrogen fertilizer levels in different growth stages in 2019 and 2020.

## Data Availability

All data supporting the conclusions of this article are provided within the article (and its additional fles).

## Abbreviations

CP: Conventional plant
AWD: Alternate wet and dry
UAV: Unmanned aerial vehicle
DSM: Digital surface model
