# Supplementary material for: Controlling the lodging risk of rice based on a plant height dynamic model
Source: Bot Stud. 2022 Aug 26;63:25. doi: 10.1186/s40529-022-00356-7 (PMC9411474; doi:10.1186/s40529-022-00356-7)
Supplement: Supplementary file 1 — Additional file1: Table S1. Plant height of TNG71 in different growth stages in 2019 and 2020. Table S2. Lodge rate, yield, and plant height of TNG71 under four nitrogen fertilizer levels in different growth stages in 2019 and 2020. [file 40529_2022_356_MOESM1_ESM.docx]

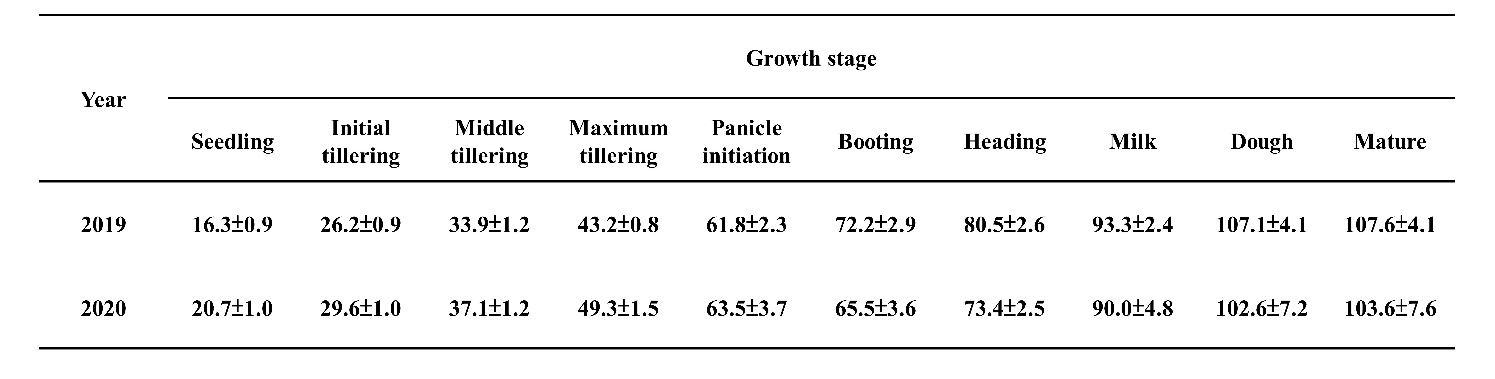
Table S1. Plant height of TNG71 in different growth stages in 2019 and 2020.


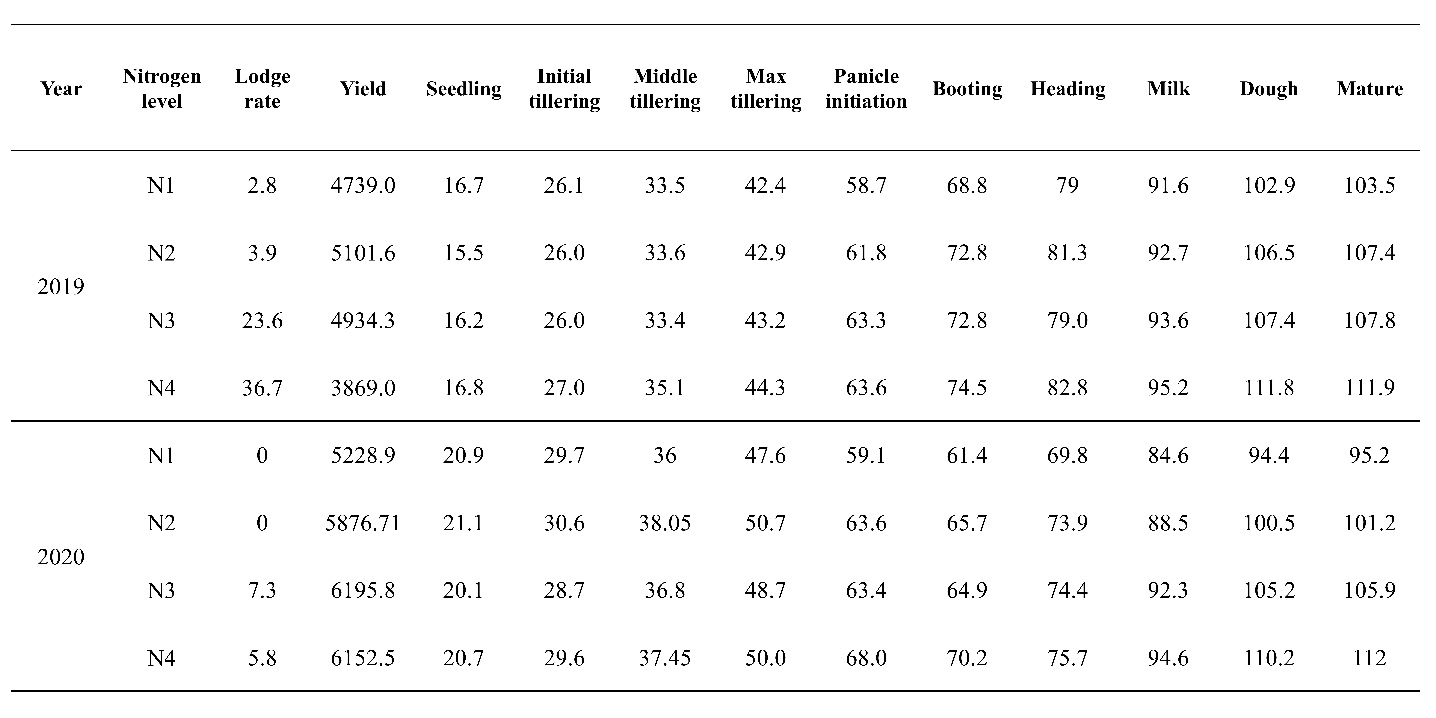
Table S2. Lodge rate, yield, and plant height of TNG71 under four nitrogen fertilizer levels in different growth stages in 2019 and 2020.
